# Obesity Severity, Dietary Behaviors, and Lifestyle Risks Vary by Race/Ethnicity and Age in a Northern California Cohort of Children with Obesity

**DOI:** 10.1155/2016/4287976

**Published:** 2016-01-14

**Authors:** Margaret C. Ford, Nancy P. Gordon, Amanda Howell, Cheryl E. Green, Louise C. Greenspan, Malini Chandra, R. Grant Mellor, Joan C. Lo

**Affiliations:** ^1^Department of Medicine, Kaiser Permanente Oakland Medical Center, Oakland, CA 94611, USA; ^2^Division of Research, Kaiser Permanente Northern California, Oakland, CA 94612, USA; ^3^Regional Health Education, The Permanente Medical Group, Oakland, CA 94612, USA; ^4^Department of Medicine, Kaiser Permanente Santa Rosa Medical Center, Santa Rosa, CA 95403, USA; ^5^Department of Pediatrics, Kaiser Permanente San Francisco Medical Center, San Francisco, CA 94115, USA; ^6^Department of Pediatrics, Kaiser Permanente Stockton Medical Center, Stockton, CA 95210, USA

## Abstract

Identification of modifiable behaviors is important for pediatric weight management and obesity prevention programs. This study examined obesogenic behaviors in children with obesity in a Northern California obesity intervention program using data from a parent/teen-completed intake questionnaire covering dietary and lifestyle behaviors (frequency of breakfast, family meals, unhealthy snacking and beverages, fruit/vegetable intake, sleep, screen time, and exercise). Among 7956 children with BMI ≥ 95th percentile, 45.5% were females and 14.2% were 3–5, 44.2% were 6–11, and 41.6% were 12–17 years old. One-quarter (24.9%) were non-Hispanic white, 11.3% were black, 43.5% were Hispanic, and 12.0% were Asian/Pacific Islander. Severe obesity was prevalent (37.4%), especially among blacks, Hispanics, and older children, and was associated with less frequent breakfast and exercise and excess screen time, and in young children it was associated with consumption of sweetened beverages or juice. Unhealthy dietary behaviors, screen time, limited exercise, and sleep were more prevalent in older children and in selected black, Hispanic, and Asian subgroups, where consumption of sweetened beverages or juice was especially high. Overall, obesity severity and obesogenic behaviors increased with age and varied by gender and race/ethnicity. We identified several key prevalent modifiable behaviors that can be targeted by healthcare professionals to reduce obesity when counseling children with obesity and their parents.

## 1. Introduction

The high prevalence of pediatric obesity remains a major healthcare burden and an area of high priority for population management in pediatric care. Given the strong tracking effect of childhood obesity into adulthood [1–4] and the association of pediatric obesity with adverse health consequences [5–7], understanding behavioral factors, severity trends, and high-risk subgroups is important from both a public health and clinical perspective. Although overall pediatric obesity rates have stabilized in recent years [8], rates of severe obesity are high [9], and the fact remains that one in six US children have obesity [8, 10–12], with the burden disproportionately affecting Hispanic/Latino and black pediatric populations [13–18]. Newer definitions for higher order obesity delineated by the body mass index (BMI) percentage above the 95th BMI percentile provide additional classification of children with severe obesity [19, 20], where a continued upward trend has been observed nationally [9] and racial/ethnic disparities are evident beginning at an early age [15, 17].

The US Preventive Services Task Force and the American Association of Pediatrics recommend BMI screening for children starting at a very young age, citing evidence for early detection and management of pediatric patients with obesity [21–23]. At a national level, pediatric weight assessment, nutrition counseling, and counseling for physical activity are also important benchmarks in clinical practice, with implementation of standards by the National Committee for Quality Assurance in 2009 [24]. In 2013, the American Heart Association [19] recommended the use of new growth charts for children with severe obesity [20], with the goal of optimizing weight classification and BMI tracking above 120% of the 95th BMI percentile [20, 25]. While demographic and behavioral factors have been well documented in pediatric overweight and obesity [26–33], fewer studies have examined differences by obesity severity using these contemporary classification criteria. Additionally, the characterizations of dietary and lifestyle behaviors at the extremes of pediatric obesity remain understudied. A better understanding of these modifiable behaviors may lead to more targeted screening for specific risk (or protective) factors that can inform the focus of targeted intervention to decrease pediatric obesity.

Kaiser Permanente Northern California (KPNC) is a large integrated health care delivery system providing comprehensive care for an ethnically diverse population of more than three million members, of whom nearly one-fourth are children under the age of 18 years. Within KPNC, a multifaceted approach to address the growing prevalence of childhood obesity was initiated in 2001, with three main components: medical office visit interventions (screening and counseling), weight management interventions, and environmental changes [13]. These efforts resulted in a modest reduction in obesity prevalence in younger children, but not adolescents, between 2003–2005 and 2009-2010 [13]. In 2012, KPNC further implemented a population program entitled the Get Healthy Action Plan (GHAP), designed to assist pediatricians in the assessment and counseling of children and adolescents with obesity using a standardized office tool during routine well-child visits. Following measurement of height and weight, children and adolescents with BMI ≥ 95th percentile were identified by medical assistants, and the child's parent or the teen patient was asked to complete a brief questionnaire focused on dietary and lifestyle behaviors. Pediatricians reviewed the responses during the visit to facilitate discussion and encourage follow-up of healthy behaviors. Within 2 years, the GHAP program identified and assessed nearly 8,000 children and adolescents who met BMI criteria for obesity during their well-child visit. As such, the GHAP well-child visit encounter provides an extremely large, diverse, and real-world clinical population of children with obesity in whom obesity severity, risk behaviors, weight status, and intervention can be further examined.

The purpose of this study was to characterize the anthropometric findings, demographic factors, and behavioral correlates of obesity severity in a racial/ethnically diverse cohort of pediatric patients evaluated in the KPNC GHAP program. In addition, we wanted to use the GHAP questionnaire data as a contemporary and complete assessment of current pediatric dietary and lifestyle behaviors and trends, identifying high risk subgroups by age, gender, race/ethnicity, and weight status for future management and tracking. It is anticipated that these findings will aid in the development of targeted interventions to address specific modifiable obesogenic dietary and lifestyle behaviors in children and adolescents who have obesity or are at risk for developing obesity.

## 2. Material and Methods

This retrospective cohort study examined demographic factors and obesity severity among children assessed through the GHAP program, a population-based intervention implemented at KPNC well-child visits that utilized a pediatrician-developed (C.G.) primary care pediatric obesity assessment tool kit with medical assistant support. The study was approved by the KPNC Institutional Review Board and a waiver of informed consent was obtained due to the nature of the study.

### 2.1. Cohort Ascertainment, Anthropometry, and Weight Tracking

The study cohort included children and adolescents 3–17.9 years old who underwent a baseline GHAP assessment at a well-child visit between January 1, 2012, and December 31, 2013. The GHAP program was piloted in 2011 in Santa Rosa (C.G.) and subsequently scaled for broader use across more than 30 medical offices in KPNC. Pediatric Weight Management Physician Champions and Chiefs of Pediatrics for Health Plan Medical Centers were briefed on the pilot program and encouraged to adopt the tools, but participation was voluntary. Typically, medical assistants would initiate the GHAP assessment for children whose BMI was ≥95th percentile for age and sex, and, less often, physicians in some sites asked parents (or teens) to complete the questionnaire mid-way through the visit. Providers then initiated their health counseling customized to the patient's answers on the questionnaire. Questionnaire responses were entered into the electronic medical record.

Administrative databases were used to obtain information on age, sex, and race/ethnicity. Census block-level data were used as a proxy for socioeconomic status, with residence in a lower income neighborhood defined as living in an area with a median household income ≤ $40,000. The electronic medical record was used to ascertain clinic-based measurements of height by stadiometer and weight using a calibrated scale. Body mass index was calculated as weight divided by height squared (kg/m^2^) and classified by BMI percentiles according to age- and gender-specific 2000 CDC growth charts [34, 35]; weight status was categorized as normal to underweight (BMI < 85th percentile), overweight (BMI 85th to <95th percentile), or obese (BMI ≥ 95th percentile) for age and sex [23]. Higher order obesity was expressed as a percentage of the 95th BMI percentile, with severe obesity defined by a threshold of BMI ≥ 120% of the 95th BMI percentile [19, 20, 25].

### 2.2. Behavior/Lifestyle Risk Factors

Data on behavioral and lifestyle risk factors came from a one-page 19-item GHAP questionnaire that was completed by parents of children with obesity and/or adolescents with obesity at the time of a well-child visit (teens were given the questionnaire directly). The GHAP form included specific questions pertaining to nine dietary/lifestyle behaviors: the number of unhealthy snacks, sugar-sweetened beverages (SSBs), and juice drinks per day; the numbers of servings of fruits and vegetables per day; the frequency of eating breakfast and family meals; the number of days per week the child was physically active for at least 60 minutes outside of school physical education; the number of hours of “screen time” (time spent playing video games, using a computer, or watching television) per day; and the number of hours of sleep per night. Inadequate sleep was defined per National Sleep Foundation 2015 Consensus Guidelines [36] which recommends against sleep less than 8 hours per day (including naps) for children aged 3–5 and less than 7 hours per day for children aged 6–17 years. To assist parents and teens with objective reporting of dietary behaviors, specific examples were given for sugar-sweetened beverages (e.g., soda, Gatorade, Vitamin Water, Hi-C, and energy drinks) and sweet and salty snacks (cookies, candy, chips, crackers, etc.), and the serving size for fruits and vegetables was characterized as 1/2 cup (or 4 ounces). Numeric response categories were provided for each question. All behavior/lifestyle risk factors were framed as unhealthy behaviors in our analyses so that a higher percentage of each behavior reflects a higher percentage of children in that group with that unhealthy behavioral or lifestyle risk. Analyses pertaining to behavior and lifestyle risks were restricted to the 98.7% of the cohort (*n* = 7,856) for whom information was available for at least five of these nine behaviors. The age, race/ethnicity, and obesity severity profile of this slightly restricted cohort did not differ from that of the full cohort.

### 2.3. Statistical Analysis

Descriptive statistics were performed using the chi-square test for categorical variables and Student's *t*-test for continuous variables (Wilcoxon rank sum test for nonparametric data). Trends in severe obesity by age group were examined using the Cochrane-Armitage test. Differences in dietary and lifestyle behaviors by age group (6–11 years and 12–17 years versus 3–5 years) and by race/ethnicity (black, Hispanic, and Asian/Pacific Islander versus non-Hispanic white) or obesity severity (BMI 100–119% of the 95th BMI percentile versus BMI ≥ 120% of the 95th BMI percentile for age and sex) within age group were examined using logistic regression models that controlled for race/ethnicity and gender (age group and obesity severity differences) or age group and gender (racial/ethnic differences). All analyses were conducted using SAS Version 9.3 (SAS Institute; Cary, NC) [37]. Comparisons were considered statistically significant based on a *p* value of <0.05 using two-tailed tests.

## 3. Results

A total of 7956 children and adolescents aged 3–17.9 years with a BMI ≥ 95th percentile had a baseline GHAP assessment at a KPNC well-child visit during 2012-2013. Nearly half of them were females (45.5%) and there was notable racial/ethnic diversity, including 24.9% non-Hispanic white (whiteNH), 11.3% black, 43.5% Hispanic, 12.0% Asian/Pacific Islander (Asian/PI), and 8.3% other/unknown race/ethnicity. Most children were 6–11 (44.2%) or 12–17 (41.6%) years old, with only 14.2% 3–5 years old. Based on residential census block data, 14.3% of these children lived in a lower income neighborhood, with Hispanic and black children two to three times as likely as whiteNH and Asian/PI children to live in a lower income neighborhood (21.8% and 19.0% versus 6.8% and 7.5%, resp., *p* < 0.001).

Table 1 shows the distribution of moderate and severe obesity by gender and race/ethnicity for preschool-aged children (3–5 years), school-aged children (6–11 years), and adolescents (12–17 years). Over one-third (37.4%) of these children with obesity met criteria for severe obesity (BMI ≥ 120% of the 95th percentile), with one-tenth found to have a BMI ≥ 140% of the 95th percentile. The proportion of children with severe obesity increased significantly by age group (31.4%, 36.1%, and 40.9%, *p* < 0.001 for trend). Across all age groups, black and Hispanic children were more likely than whiteNH children to meet criteria for severe obesity (ages 3–5, 37.6% and 34.0% versus 24.8%, resp.; ages 6–11, 43.9% and 40.1% versus 28.9%; and ages 12–17, 50.5% and 43.2% versus 37.3%, all *p* < 0.05). Asian/PI children in the youngest group were more likely than whiteNH children to have severe obesity (38.8% versus 24.8%, *p* < 0.01), but rates did not differ significantly from whiteNHs in the older groups. Preschool-aged children who lived in a lower income neighborhood were also more likely than those who did not to have severe obesity (39.3% versus 29.7%, *p* < 0.05 after adjusting for race/ethnicity and gender); however, living in a lower income neighborhood was not associated with obesity severity among older children.

Table 2 profiles obesity-related behaviors and lifestyle risks of children in each of the three age groups (statistical modeling found few differences in behaviors by gender, and, where differences exist, they are noted in the text). Compared to the youngest children, those in the two older age groups had higher percentages of obesogenic health behaviors, including being more likely to eat breakfast <6 days per week, to eat meals with their family <5 times per week, to eat ≥3 sugary or salty snacks per day, to eat <5 servings of fruits and/or vegetables per day, to get <5 days of exercise (at least 60 minutes outside of school) per week, and to have “screen time” ≥ 3 hours per day. Consumption of ≥2 SSBs/juice drinks per day did not differ by age, as rates were already extremely high (58%) among preschool-aged children.

Few gender differences in dietary and lifestyle risks were observed within each age group. In the youngest age group, girls were more likely than boys to have <5 days of exercise (at least 60 minutes outside of school) per week (47.7% versus 41.6%) but tended to be less likely to have ≥3 hours of “screen time” (28.7% versus 34.0%). In the 6- to 11-year-old age group, girls were less likely than boys to have ≥3 sugary or salty snacks per day (26.0% versus 29.8%), to have ≥2 SSBs/juice drinks per day (55.6% versus 62.6%), and to have ≥3 hours of “screen time” per day (40.4% versus 45.9%), but they were more likely to have <5 days of exercise (at least 60 minutes outside of school) per week (68.2% versus 62.3%). In the adolescent age group, girls were more likely than boys to eat breakfast <6 times per week (63.0% versus 55.4%), but they were less likely to consume ≥2 SSBs/juice drinks per day (57.0% versus 62.2%). They were also more likely than boys to have <5 days of exercise (at least 60 minutes outside of school) per week (74.8% versus 61.8%) and to have no days of exercise (17.7% versus 9.6%). Adolescent girls were nearly twice as likely as boys to get <7 hours of sleep per night (11.7% versus 6.7%). Fruit and vegetable intake also varied by gender among younger children. These differences remained significant after adjusting for race/ethnicity.

Table 3 examines how these health-related behaviors/lifestyle risks differ by obesity severity within each age group, using moderate obesity as the reference group. Among preschool-aged children, a higher percentage of children with severe obesity ate breakfast <6 times per week, consumed ≥2 SSBs/juice per day, and had <5 days of exercise (at least 60 minutes outside school) per week. School-aged children with severe obesity were also more likely to eat breakfast <6 times per week and have <5 days of exercise (at least 60 minutes outside school) per week, in addition to limited family meals and greater “screen time.” Adolescents with severe obesity shared these same unhealthy behavior trends, along with lower fruit/vegetable intake and inadequate sleep, but there was no difference in the proportion eating regular family meals when compared to adolescents with moderate obesity. These behavioral differences by obesity severity persisted after additionally controlling for residence in a lower income neighborhood (data not shown).

Significant racial/ethnic differences in obesogenic health behavior and lifestyle risks are identified in Table 4. Black and Hispanic children of all age groups and younger Asian/PI children were more likely than whiteNH children to drink ≥2 SSBs/day. Selected age subsets of black and Asian/PI children were also more likely than whiteNH children to consume ≥3 sugary or salty snacks a day. Hispanic children and older black children were more likely than whiteNH children to eat breakfast <6 times a week and have family meals <5 days per week. Exercise level varied by race/ethnicity within age groups (consistently lower in Hispanic and younger Asian/PI children compared to whiteNH children), with low rates of exercise overall. More than half of 6–11 year olds and two-thirds of 12–17 year olds across all racial/ethnic subgroups reported getting <5 days of exercise (at least 60 minutes outside school) per week, with large percentages getting <3 days of exercise per week. Black children across all age groups and Asian/PI and Hispanic children among selected younger age groups were more likely than whiteNH children to have ≥3 hours of screen time per day; nearly two-thirds of adolescents in all racial/ethnic subgroups reported ≥3 hours of screen time per day. Sleep behavior also differed, with significantly greater proportion of black and Hispanic adolescents and preschool-aged black children reporting inadequate hours of sleep per night. These racial/ethnic differences persisted after additionally controlling for residence in a lower income neighborhood (data not shown).

## 4. Discussion

We found that, in a racially and ethnically diverse outpatient pediatric population with moderate or severe obesity, a clinical screening questionnaire completed by parents of children with obesity or teens with obesity in the context of a well-child visit identified large numbers of preschool-aged, school-aged, and adolescent children with modifiable obesity-related dietary and lifestyle risk factors. In addition to confirming substantial opportunities for intervention with education pertaining to increased exercise and reduced screen time, SSB/juice intake, and unhealthy snack consumption, we also found that large numbers of children with obesity were not regularly having breakfast or family meals. Notably, these adverse health behaviors started at a very early age and many increased with level of BMI. The high rates of excess SSB/juice intake among preschool children (nearly two-thirds among those with severe obesity) support data from other studies demonstrating high consumption of sweetened beverages and association with adiposity in preschool-aged children [38–40] and underscore the need for early education and counseling of parents and caregivers [38]. Disparities by race/ethnicity were also seen, with greater numbers of unhealthy behaviors exhibited by young black, Hispanic, and Asian/PI children as compared to whiteNH children. Among adolescents, the largest differences were seen in black and/or Hispanic populations, where unhealthy behaviors were significantly more prevalent than in whiteNH children. Nearly two-thirds and three-fourths of black and Hispanic school-aged children and adolescents reported excess consumption of SSB/juices on a daily basis. These and other modifiable behaviors present important intervention targets for counseling during well-child visits, with tracking of behavior change through follow-up. The GHAP program also provides a future platform for examining less modifiable factors, including financial and logistical barriers, as well as broader issues pertaining to health literacy that can be addressed through additional GHAP surveys.

Our findings are consistent with data from other studies which show higher consumption of sugary drinks [29, 41]; more hours of weekday television, lower participation in vigorous exercise, and lower proportion eating breakfast among black and Hispanic children compared to whiteNH children [27, 30, 33], as well as higher percentages with low socioeconomic status [28, 30]. Children with low socioeconomic status have also been found to have more obesogenic behaviors [28, 30]. In our current study, obesogenic behaviors and lifestyle factors varied by obesity severity and race/ethnicity, independent of residence in a lower income neighborhood. While further examination of socioeconomic influences is warranted, these data may help direct larger sociopolitical agendas pertaining to food policy, education, school-based initiatives, and environmental influences to address challenges faced at the individual patient level.

While severe obesity and unhealthy behaviors were highly prevalent in black and Hispanic subgroups, we also found gender differences that persisted after controlling for race/ethnicity. For example, insufficient exercise was a consistent unhealthy behavior with higher prevalence among school-aged and adolescent girls compared to boys, in addition to lower frequency of daily breakfast and inadequate hours of sleep per night among adolescent girls. The high proportion of adolescents with excessive “screen time” poses an additional challenge, given that, in addition to watching television, playing video games, and using the Internet and online social media, adolescents may be required to spend time on the computer to complete homework. While these self-reported behaviors should be differentiated for counseling purposes, collectively they represent unhealthy sedentary behaviors that could contribute to obesity risk. The model of GHAP, which elegantly structures the conversation between the primary care physician and the patient and family, allows the conversation with families and teens to focus on individual challenges with regard to healthy behaviors, while lessening the tendency towards weight bias and stigma.

There are some limitations of our study to consider. First, the GHAP questionnaire responses were based on self-reported behaviors by the parent or teen, potentially subject to overreporting of healthy behaviors and underreporting of unhealthy behaviors. Future validation efforts could include a diet/physical activity log for comparison. Second, the GHAP program was restricted to children with obesity and thus these behaviors could not be compared to those of nonobese peers, including differences by race/ethnicity. However, our goal was to examine modifiable risk behaviors among children with obesity for potential intervention. Third, since these data are cross-sectional, the results can only be interpreted as associations without inferring causality. We also acknowledge that there are “less controllable” causes of obesity, including genetics, biology, and environmental factors, not addressed in the current study. Fourth, we were not able to assign socioeconomic status based on household income or parent education and had to rely on residential census block data to characterize the neighborhoods in which the children resided. Finally, while the cohort was large, it was not large enough to examine racial/ethnic and gender differences by level of obesity within each age group, which should be examined in future studies.

The strengths of our study include the comprehensive assessment of dietary and lifestyle behaviors at a well-child visit (providing a convenient benchmark for future interventions and tracking) and the ability to examine trends in obesogenic behaviors by age, race/ethnicity, and obesity severity using contemporary weight classification criteria. Knowing these trends in unhealthy obesogenic behaviors will be useful for addressing modifiable risk factors in weight management, with behavioral intervention targets in the clinical setting particularly applicable to young children who already receive frequent routine pediatric care. Furthermore, this clinic-based population study was not restricted to research subjects or a clinical trial cohort, providing an extremely large and contemporary pediatric clinic population for which future interventions can be implemented. Our study examined a racially/ethnically and socioeconomically diverse pediatric patient population drawn from more than 30 health plan clinics across a large and varied geographic area in Northern California.

In conclusion, this study provides a contemporary assessment of behavioral trends in pediatric obesity and initial data for investigating further associations between severe and/or extreme obesity and lifestyle in children and adolescents. Similar to our prior studies conducted within the KPNC pediatric population, we found that severe obesity rates were high among the subgroup of black and Hispanic children with obesity [15, 42], beginning at an early age. Given the strong tracking effect for both moderate and severe obesity in childhood [42], these observations underscore the need to address existing ethnic disparities and to identify young and preteen children at highest risk for sustained obesity with focus on both primary prevention and early intervention. Findings from this study will inform the development and implementation of cost-effective, targeted healthy lifestyle interventions focused on reducing obesity rates among pediatric patients in the primary care setting.

## Figures and Tables

**Table 1:** Demographic characteristics of the pediatric GHAP cohort (row percentages provided).

Age group	*N*	Moderate obesity	Severe obesity	
		BMI 100–119% of 95th percentile	BMI 120–139% of 95th percentile	BMI ≥ 140% of 95th percentile
*Ages 3–17 years*				
Gender, %				
All	7956	4978 (62.6%)	2134 (26.8%)	844 (10.6%)
Male	4333	2594 (59.9%)	1232 (28.4%)	507 (11.7%)
Female	3623	2384 (65.8%)	902 (24.9%)	337 (9.3%)
Race/ethnicity, %				
WhiteNH	1984	1345 (67.8%)	472 (23.8%)	167 (8.4%)
Black	899	485 (53.9%)	265 (29.5%)	149 (16.6%)
Hispanic	3457	2061 (59.6%)	1002 (29.0%)	394 (11.4%)
Asian/PI	953	632 (66.3%)	246 (25.8%)	75 (7.9%)
Other/unknown	663	455 (68.6%)	149 (22.5%)	59 (8.9%)
Lower income neighborhood, %				
Yes	1139	671 (58.9%)	326 (28.6%)	142 (12.5%)
No	6817	4307 (63.2%)	1808 (26.5%)	702 (10.3%)

*Ages 3–5 years*				
Gender, %				
All	1130	775 (68.6%)	265 (23.4%)	90 (8.0%)
Male	573	390 (68.1%)	133 (23.2%)	50 (8.7%)
Female	557	385 (69.1%)	132 (23.7%)	40 (7.2%)
Race/ethnicity, %				
WhiteNH	250	188 (75.2%)	50 (20.0%)	12 (4.8%)
Black	117	73 (62.4%)	32 (27.3%)	12 (10.3%)
Hispanic	527	348 (66.0%)	128 (24.3%)	51 (9.7%)
Asian/PI	134	82 (61.2%)	39 (29.1%)	13 (9.7%)
Other/unknown	102	84 (82.3%)	16 (15.7%)	2 (2.0%)
Lower income neighborhood, %				
Yes	206	125 (60.7%)	62 (30.1%)	19 (9.2%)
No	924	650 (70.3%)	203 (22.0%)	71 (7.7%)

*Ages 6–11 years*				
Gender, %				
All	3516	2247 (63.9%)	954 (27.1%)	315 (9.0%)
Male	1928	1175 (60.9%)	553 (28.7%)	200 (10.4%)
Female	1588	1072 (67.5%)	401 (25.3%)	115 (7.2%)
Race/ethnicity, %				
WhiteNH	830	590 (71.1%)	185 (22.3%)	55 (6.6%)
Black	376	211 (56.1%)	121 (32.2%)	44 (11.7%)
Hispanic	1579	946 (59.9%)	469 (29.7%)	164 (10.4%)
Asian/PI	439	296 (67.4%)	116 (26.4%)	27 (6.2%)
Other/unknown	292	204 (69.9%)	63 (21.6%)	25 (8.6%)
Lower income neighborhood, %				
Yes	495	299 (60.4%)	145 (29.3%)	51 (10.3%)
No	3021	1948 (64.5%)	809 (26.8%)	264 (8.7%)

*Ages 12–17 years*				
Gender, %				
All	3310	1956 (59.1%)	915 (27.6%)	439 (13.3%)
Male	1832	1029 (56.2%)	546 (29.8%)	257 (14.0%)
Female	1478	927 (62.7%)	369 (25.0%)	182 (12.3%)
Race/ethnicity, %				
WhiteNH	904	567 (62.7%)	237 (26.2%)	100 (11.1%)
Black	406	201 (49.5%)	112 (27.6%)	93 (22.9%)
Hispanic	1351	767 (56.8%)	405 (30.0%)	179 (13.2%)
Asian/PI	380	254 (66.8%)	91 (24.0%)	35 (9.2%)
Other/unknown	269	167 (62.1%)	70 (26.0%)	32 (11.9%)
Lower income neighborhood, %				
Yes	438	247 (56.4%)	119 (27.2%)	72 (16.4%)
No	2872	1709 (59.5%)	796 (27.7%)	367 (12.8%)

WhiteNH = non-Hispanic white; Asian/PI = Asian or Pacific Islander; Lower income neighborhood = residing in a residential census block with a median household income ≤ $40,000.

**Table 2:** Health-related behaviors and lifestyle risks of pediatric patients with obesity, by age group.

Health behavior/lifestyle risk	Age group		
	3–5 years	6–11 years	12–17 years
	(*N* = 1115)	(*N* = 3484)	(*N* = 3257)
	%	%	%
Eating breakfast <6 days a week	28.9	34.1^a^	58.8^a^
Eating meals with family <5 times a week	34.9	39.4^a^	54.2^a^
Eating <5 servings of fruit/vegetables a day	45.1	54.8^a^	61.6^a^
Eating <3 servings of vegetables a day	60.2	65.6^a^	70.9^a^
Eating <3 servings of fruit a day	44.0	55.2^a^	63.7^a^
Eating ≥3 sugary or salty snacks a day	24.1	28.1^a^	31.9^a^
Drinking ≥2 sugary or juice drinks a day	58.0	59.4	59.8
60 min exercise outside PE <5 times a week	44.6	65.0^a^	67.6^a^
60 min exercise <3 times a week	16.6	28.6^a^	36.9^a^
TV, computer, or video games ≥3 hours a day	31.4	43.4^a^	63.5^a^
Inadequate number of hours of sleep per night^1^	6.7	1.2^a^	8.9^a^

PE = school-based physical education or recess; TV = television. Column *N*s are approximate due to missing data.

^1^The National Sleep Foundation, 2015, consensus guidelines [36] recommend against sleep <8 hours per day (including naps) for children aged 3–5 and <7 hours per day for children aged 6–17 years; these thresholds were used to define inadequate number of hours of sleep per night.

^a^Significantly (*p* < 0.05) different from ages 3–5 after adjusting for child's sex and race/ethnicity.

**Table 3:** Health-related behavior and lifestyle risks of pediatric patients with obesity, by age group and obesity severity.

Health behavior/lifestyle risk	3–5 years		6–11 years		12–17 years	
	Moderate obesity (*N* = 762) %	Severe obesity (*N* = 353) %	Moderate obesity (*N* = 2224) %	Severe obesity (*N* = 1260) %	Moderate obesity (*N* = 1916) %	Severe obesity (*N* = 1341) %
Eating breakfast <6 times a week	25.5	36.5^a^	31.2	39.3^a^	55.0	64.2^a^
Eating meals with family <5 times a week	35.4	33.8	37.6	42.5^a^	53.1	55.9
Eating <5 servings of fruits/vegetables a day	45.2	45.0	54.7	54.9	59.6	64.5^a^
Eating <3 servings of vegetables a day	60.8	59.0	65.9	65.1	69.0	73.7^a^
Eating <3 servings of fruit a day	43.1	45.9	54.4	56.5	62.5	65.4
Eating ≥3 sugary or salty snacks a day	22.9	26.8	26.7	30.5	31.1	33.1
Drinking ≥2 sugary or juice drinks a day	54.6	65.2^a^	57.4	63.0	58.4	61.9
60 min exercise outside PE <5 times a week	40.7	53.0^a^	63.0	68.5^a^	66.5	69.1^a^
60 min exercise <3 times a week	15.1	19.8	25.7	33.9^a^	34.7	40.0^a^
TV, computer, or video games ≥3 hours/day	29.5	35.4	40.5	48.6^a^	61.7	66.1^a^
Inadequate number of hours of sleep/night^1^	5.4	9.4^a^	1.0	1.5	7.5	10.9^a^

PE = school-based physical education or recess; TV = television. Column *N*s are approximate due to missing data.

^1^Sleep <8 hours per day (including naps) for ages 3–5 and <7 hours per night for ages 6–17.

^a^Significantly (*p* < 0.05) different from moderate obesity after adjusting for child's sex and race/ethnicity.

Moderate obesity = BMI 100–119% of the 95th percentile; Severe obesity = BMI ≥ 120% of the 95th percentile.

**Table 4:** Health-related behaviors and lifestyle risks of pediatric patients with obesity, by age group and race/ethnicity.

Health behavior/lifestyle risk	3–5 years				6–11 years				12–17 years			
	WhiteNH (*N* = 248) %	Black (*N* = 116) %	Hispanic (*N* = 519) %	Asian/PI (*N* = 134) %	WhiteNH (*N* = 820) %	Black (*N* = 376) %	Hispanic (*N* = 1561) %	Asian/PI (*N* = 437) %	WhiteNH (*N* = 889) %	Black(*N* = 401) %	Hispanic(*N* = 1331) %	Asian/PI (*N* = 376) %
Eating breakfast <6 times a week	19.7	25.0	33.3^a^	37.6^a^	21.4	35.2^a^	39.5^a^	35.5^a^	48.2	70.4^a^	62.8^a^	56.0^a^
Eating meals with family <5 times a week	27.9	36.3	39.5^a^	30.6	28.6	55.4^a^	41.0^a^	39.2^a^	45.6	67.4^a^	57.5^a^	48.0
Eating <5 servings of fruits/vegetables a day	37.1	38.8	49.0^a^	52.2^a^	52.8	48.3	55.5	58.0	59.4	60.3	63.9^a^	62.5
Eating <3 servings of vegetables a day	58.1	56.1	60.7	61.2	66.3	58.0^a^	67.2	64.3	70.4	66.0	74.2	67.2
Eating <3 servings of fruit a day	35.2	41.4	47.6^a^	51.9^a^	52.3	55.3	54.2	59.4^a^	62.5	65.2	65.0	60.9
Eating ≥3 sugary or salty snacks a day	20.7	22.6	23.1	33.8^a^	23.6	36.6^a^	27.3	29.6^a^	31.3	38.2^a^	29.8	30.7
Drinking ≥2 sugary or juice drinks a day	44.0	67.2^a^	64.3^a^	57.1^a^	43.1	76.9^a^	64.6^a^	57.0^a^	49.4	76.8^a^	65.0^a^	50.1
60 min exercise outside PE <5 times a week	35.1	50.9^a^	47.0^a^	50.8^a^	55.6	58.7	67.5^a^	76.1^a^	65.1	68.1	70.1^a^	67.5
60 min exercise <3 times a week	10.5	14.0	19.1^a^	23.5^a^	20.8	28.0^a^	29.7^a^	40.1^a^	33.8	39.8	39.3^a^	34.4
TV, computer, or video games ≥3 hours/day	23.8	43.5^a^	30.2	36.6^a^	37.5	46.2^a^	43.6^a^	49.5^a^	62.1	72.0^a^	61.4	63.9
Inadequate number of hours of sleep/night^1^	4.5	17.0^a^	6.3	5.2	1.4	2.1	1.0	0.7	7.0	12.2^a^	9.7^a^	6.2

WhiteNH = non-Hispanic white; Asian/PI = Asian or Pacific Islander. PE = school-based physical education or recess; TV = television. Column *N*s are approximate due to missing data.

^1^Sleep <8 hours per day (including naps) for ages 3–5 and <7 hours per night for ages 6–17.

^a^Significantly (*p* < 0.05) different from non-Hispanic whites after adjusting for child's sex.
